# Systemic Exposure to Air Pollution Induces Oxidative Stress and Inflammation in Mouse Brain, Contributing to Neurodegeneration Onset

**DOI:** 10.3390/ijms21103699

**Published:** 2020-05-24

**Authors:** Chiara Milani, Francesca Farina, Laura Botto, Luca Massimino, Elena Lonati, Elisabetta Donzelli, Elisa Ballarini, Luca Crippa, Paola Marmiroli, Alessandra Bulbarelli, Paola Palestini

**Affiliations:** 1 School of Medicine and Surgery, University of Milano-Bicocca, 20900 Monza, Italy; francesca.farina@hotmail.it (F.F.); laura.botto@unimib.it (L.B.); elena.lonati1@unimib.it (E.L.); elisabetta.donzelli@unimib.it (E.D.); elisa.ballarini@unimib.it (E.B.); luca.crippa@unimib.it (L.C.); alessandra.bulbarelli@unimib.it (A.B.); paola.palestini@unimib.it (P.P.); 2 NeuroMi, Milan Centre for Neuroscience, University of Milano-Bicocca, 20900 Monza, Italy; paola.marmiroli@unimib.it; 3 POLARIS Research Centre, University of Milano-Bicocca, 20126 Milan, Italy; 4 Division of Neuroscience, San Raffaele scientific institute, 20121 Milan, Italy; admin@lucamassimino.com; 5 Department of Biotechnology and Biosciences, University of Milano-Bicocca, 20126 Milan, Italy

**Keywords:** biomass combustion, diesel exhaust, intratracheal instillation, oxidative stress, inflammation, amyloid precursor protein

## Abstract

In northern Italy, biomass burning-derived (BB) particles and diesel exhaust particles (DEP) are considered the most significant contributors to ultrafine particle (UFP) emission. However, a comparison between their impact on different brain regions was not investigated until now. Therefore, male BALB/c mice were treated with a single or three consecutive intratracheal instillations using 50 µg of UFPs in 100 µL of isotonic saline solution or 100 µL of isotonic saline solution alone, and brains were collected and analyzed. Proteins related to oxidative stress and inflammation, as well as Alzheimer’s disease markers, were examined in the hippocampus, cerebellum, and the rest of the brain (RoB). Histopathological examination of the brain was also performed. Moreover, correlations among different brain, pulmonary, and cardiovascular markers were performed, allowing us to identify the potentially most stressful UFP source. Although both acute exposures induced inflammatory pathways in mouse brain, only DEP showed strong oxidative stress. The sub-acute exposure also induced the modulation of APP and BACE1 protein levels for both UFPs. We observed that DEP exposure is more harmful than BB, and this different response could be explained by this UFP’s different chemical composition and reactivity.

## 1. Introduction

Airborne particulate matter (PM) is a complex mixture of solid and liquid particles with different size, composition, and origin, suspended in the air [1]. Recently, ultrafine particles (UFPs, with aerodynamic diameter (d_a_) < 0.1 μm) received increased attention, as they are ubiquitous in the ambient air (both indoor and outdoor), originating from many anthropogenic and natural sources [2,3], although they derive primarily from combustion processes in urban settings [4]. The toxic potential of UFPs on human health is due to different characteristics such as small size, high surface-to-volume ratio, prolonged residence time in the lungs because of low clearance efficiency, and the possibility to translocate across epithelial/endothelial barriers into the blood and lymphatic circulation [5]. 

In Lombardy (northern Italy), solid biomass burning for domestic heating and diesel combustion used for private and public transport are estimated to be the major sources of fine particle emissions (PM_2.5_, d_a_ < 2.5 µm), accounting for 49.8% and 21.5%, respectively [6,7,8]. However, biomass burning and diesel combustion mainly produce particles of 15–30 nm in diameter, often aggregated; therefore, these processes are considered significant contributors to UFP emission [9,10]. 

During the last few years, the use of biomass burning for residential heating experienced a substantial increase in northern Italy and Europe as a result of the affordable price of solid biomass fuels and as a consequence of the policy that aims to reduce greenhouse gases (EU Directive, 2009/287/EC). Unfortunately, traditional domestic appliances, still very widespread, have generally lower combustion efficiency compared to new-generation boilers, which show decreased emissions determined by improved combustion technologies [10,11]. The result is a relatively high UFP emission, which contains a multitude of toxic or carcinogenic compounds, including free radicals, polycyclic aromatic hydrocarbons (PAHs), and aldehydes [12]. 

Diesel exhaust is a complex mixture of solid, condensed, and gaseous fractions [13]. During the last few decades, it received huge attention by toxicologists, since about 18,000 high-molecular-weight organic compounds can be adsorbed on its carbonic nucleus [13,14], leading to different health effects [15]. Noteworthy, in 2012, the International Agency for Research on Cancer [16] classified diesel engine exhaust as Group 1, carcinogenic to humans, based on “sufficient evidence that exposure is associated with an increased risk for lung cancer” [16].

Moreover, acute and chronic exposure to UFPs are associated with different health effects, including nose and eye irritation, lung function impairment, headache, fatigue, nausea, and profound inflammatory changes in the airways [15]. Today, it is well established that UFPs can reach and act on different extra pulmonary organs and tissues, including the central nervous system (CNS) [17]. In fact, owing to their small size, UFPs could avoid normal phagocytic defenses in the respiratory system, crossing both the blood–air barrier and the blood–brain barrier (BBB), gaining access to the CNS [18,19,20,21]. In humans, the literature on particle translocation is still conflicting, and the amount of UFPs that translocate into blood and extra pulmonary organs varies among the different studies [19,22,23,24]. However, it is hypothesized that UFP translocation could be possible as naked particles, taken up by erythrocytes [25], or after ingestion by alveolar macrophages (AMs) [26]. In addition, UFPs can reach the CNS through the olfactory mucosa, the neuronal epithelium that is in direct contact with the environmental air [20,24,27]. Finally, inhaled particles may affect the CNS and other tissues through systemic inflammatory mediators (e.g., interleukin-6 (IL-6), tumor necrosis factor (TNF-α)), histamine, and oxidative stress products) produced in the lungs because of chronic pollutant-induced epithelial and endothelial injury and released into the circulation [3,20]. Importantly, even though the translocation rate of UFPs from their entry site to secondary organs might be rather low, continuous or chronic exposure to air pollution may result in the accumulation of toxic molecules in the brain as a secondary target organ in significant amounts [20,28]. 

Currently, due to their physicochemical characteristics, UFPs are known to affect the CNS via a range of cellular, molecular, and inflammatory pathways that directly damage brain structures predisposing to neurological disorders such as Alzheimer’s disease (AD) [20,29]. Oxidative stress and neuroinflammation are thought to be two cardinal processes of brain damage after UFP exposure [30]. 

Despite the increasing evidence underlying specific health effects of particles with different origins [10,31,32,33], the literature is still lacking comparative in vivo studies focused on the brain. Therefore, in the current study, male BALB/c mice were selected as an in vivo model to investigate if the same dose of UFPs derived from different anthropogenic sources (biomass burning-derived particles, BB, and diesel exhaust particles, DEP) administered by means of the same procedure (i.e., intratracheal instillation) could differently affect brain regions. Single exposures were performed in order to disclose the short-term impact of UFPs, mostly related to oxidative stress and inflammation, while three repeated instillations in a week were carried out to understand if the effect of the single instillation is amplified when repeated or if a normal condition is restored. Specifically, markers of oxidative stress (heme oxygenase-1 (HO-1), heat-shock protein 70 (Hsp70), and Cytochrome P450 1b1 (Cyp1b1)) and inflammation (inducible nitric oxide synthase (iNOS) and cyclooxygenase 2 ( COX-2)) were considered. Moreover, to further investigate brain inflammation, sham and treated mice were subjected to fluorescence molecular tomography. Finally, considering the correlation between neuroinflammation, oxidative stress, and AD [34,35], after UFP exposure, we analyzed proteins involved in amyloidogenic processing (amyloid precursor protein (APP), phosphorylated APP on threonine 668 (p-APP ^Thr668^), and beta-secretase 1 (BACE1)) as markers of neurodegeneration. Statistical correlations among different brain regions, and between brain, pulmonary, and cardiovascular markers (analyzed in the same mice and presented in Reference [33]) were performed, allowing us to identify the potentially most stressful UFP source.

## 2. Results

### 2.1. BB and DEP Exposure Does Not Affect Brain Morphology

To investigate the occurrence of brain alterations following UFP exposure, histological examinations of cerebellum, cerebral cortex, and hippocampus were performed on sham, as well as BB- and DEP-treated mice, after single and repeated instillations. 

Histological analysis performed on treated and sham mice did not show significant changes in gray and white matter in all three brain areas examined. Neurons displayed normal appearance and regular arrangement in the cerebellum, cerebral cortex, and hippocampus. No infiltration of inflammatory cells was detected in BB- and DEP-exposed mice, as well as in controls. Brain appearance was normal in all areas examined either after single or repeated instillations (Figures S1 and S2, Supplementary Materials).

### 2.2. Induction of Oxidative Stress Related Proteins under BB and DEP Exposure

To test the ability of particles to stimulate oxidative stress-related pathways, HO-1, Hsp70, and Cyp1b1 protein levels were analyzed in sham, as well as BB- and DEP-treated mice, after single or repeated instillations. 

**Figure 1:**
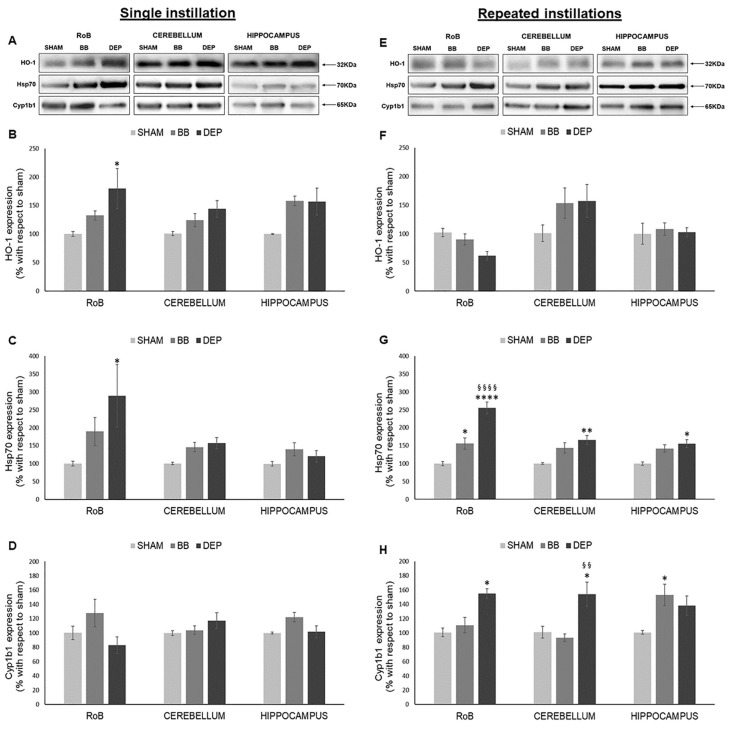
Oxidative stress analysis after single and repeated instillations of biomass burning-derived (BB) particles and diesel exhaust particles (DEP). Representative immunoblotting images of heme oxygenase-1 (HO-1), heat-shock protein 70 (Hsp70), and Cytochrome P450 1b1 (Cyp1b1) analysis in mice after single (**A**) and repeated (**E**) instillations with 50 µg of BB or DEP/100 µL 0.9% NaCl. Histograms display HO-1, Hsp70, and Cyp1b1 expression in mice after single (**B**–**D**) and repeated (**F**–**H**) instillations with BB and DEP, with respect to sham. Proteins are normalized to corresponding total proteins revealed by Ponceau in each lane (Figure S3, Supplementary Materials), and the data are expressed as means ± standard error of the mean (SEM) (*n* = 6). Statistical differences were tested accordingly by one-way ANOVA followed by Tukey post hoc comparison. * *p* < 0.05 vs. sham mice; ** *p* < 0.01 vs. sham mice, **** *p* < 0.0001 vs. sham mice; §§ *p* < 0.01 vs. BB-treated mice; §§§§ *p* < 0.0001 vs. BB-treated mice.

As shown in Figure 1, HO-1 significantly increased only after DEP single instillation in the rest of the brain (RoB) (+79.45% ± 35.22%), instead showing increasing trends in the cerebellum and hippocampus of DEP-treated mice and in all brain regions of BB-treated mice. On the other hand, HO-1 showed no significant variations after repeated exposure (Figure 1A,B,E,F).

After a single instillation, DEP treatment caused a significant increase in Hsp70 expression in the RoB (+189.64% ± 87.52%). This increase was maintained in the RoB (+155.46% ± 16.27%) after repeated exposure and was also observed in the cerebellum (+65.30% ± 12.66%) and hippocampus (+54.81% ± 11.01%), when compared to sham. BB produced a general increasing trend of Hsp70 levels, which resulted in it being significant only in the RoB after repeated instillations (+55.79% ± 15.88%) (Figure 1A,C,E,G).

Furthermore, after a single BB or DEP instillation, Cyp1b1 protein levels showed no variations in all the different brain regions. Conversely, repeated DEP exposure induced a significant increase in Cyp1b1 expression in the RoB (+55.37% ± 6.56%) and cerebellum (+53.31% ± 16.92%), while repeated BB treatment caused an increase in Cyp1b1 protein levels in the hippocampus (+52.48% ± 14.85%), when compared to sham (Figure 1A,D,E,H).

### 2.3. Induction of Inflammation-Related Proteins under BB and DEP Treatment 

The iNOS and COX-2 protein levels were analyzed in sham and treated mice to study the potential involvement of an inflammatory response.

**Figure 2:**
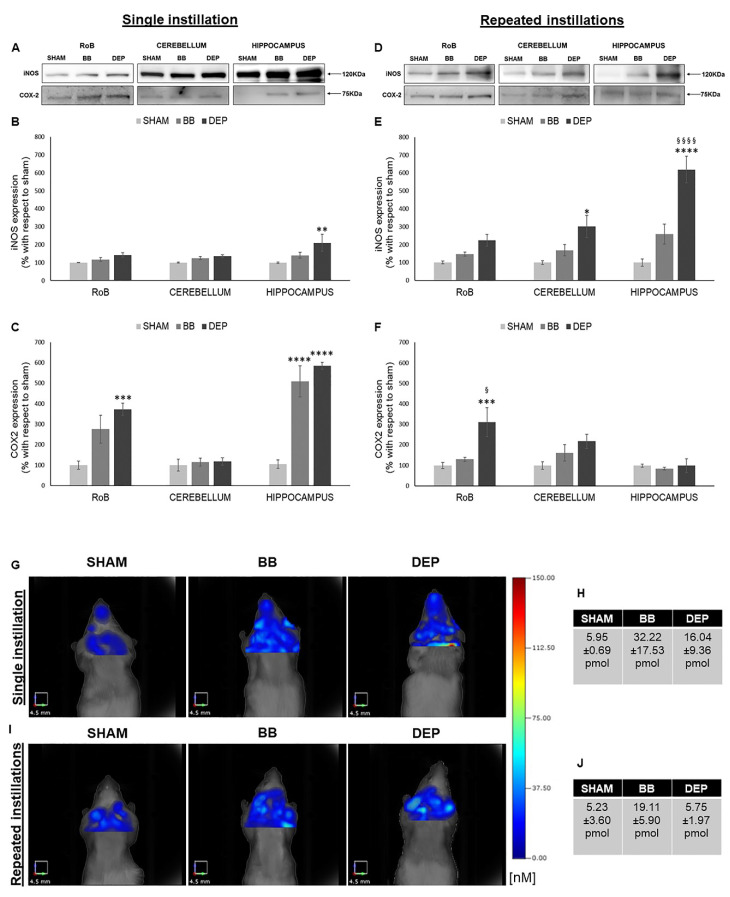
Inflammation analysis after single and repeated instillations of BB and DEP. (**A**–**F**) Representative immunoblotting images of inducible nitric oxide synthase (iNOS) and cyclooxygenase 2 ( COX-2) analysis in mice after single (**A**) and repeated (**D**) instillations with 50 µg of BB or DEP/100 µL 0.9% NaCl. Histograms display iNOS and COX-2 expression in mice after single (**B**,**C**) and repeated (**E**,**F**) instillations with BB and DEP, with respect to sham. Proteins are normalized to corresponding total proteins revealed by Ponceau in each lane (Figure S3, Supplementary Materials), and the data are expressed as means ± SEM (*n* = 6). Statistical differences were tested accordingly by one-way ANOVA followed by Tukey post hoc comparison. * *p* < 0.05 vs. sham mice; ** *p* < 0.01 vs. sham mice; *** *p* < 0.001 vs. sham mice; **** *p* < 0.0001 vs. sham mice; § *p* < 0.05 vs. BB-treated mice; §§§§ *p* < 0.0001 vs. BB-treated mice. (**G**–**J**) Representative fluorescence molecular tomography (FMT) images of sham, as well as BB- and DEP-treated, mouse brain obtained 24 h after single (**G**) and repeated (**I**) intratracheal instillations with 50 µg of BB or DEP/100 µL 0.9% NaCl. Each figure represents the results obtained from two mice for every treatment, and tables report the quantification of MMPsenseTM 750 FAST probe (pmol) after single (**H**) and repeated (**J**) intratracheal instillations. Data are expressed as means ± standard deviation.

As reported in Figure 2, single DEP treatment caused a significant increase in iNOS expression in the hippocampus (+110.70% ± 47.25%), showing increasing trends in the RoB and cerebellum. This increasing trend was maintained during repeated DEP exposure (+123.49% ± 32.05% in the RoB and +201.29% ± 61.42% in the cerebellum); in particular, we observed a huge rise of iNOS protein levels in the hippocampus (+518.51% ± 74.03%), which was statistically significant when compared to both sham and BB. Moreover, BB treatment induced a general iNOS expression increasing trend (Figure 2A,B,D,E).

Furthermore, both UFP single instillations caused an increase in COX-2 protein levels in the RoB (+175.18% ± 68.42% with BB and + 272.34% ± 29.53% with DEP), which resulted highly significant in the hippocampus (+403.86% ± 76.34% with BB and +480.18% ± 16.69% with DEP). After repeated instillations, no changes in BB-treated mice were detected, while DEP produced a significant increase in COX-2 expression in the RoB (+211.77% ± 71.47%), compared to both sham and BB (Figure 2A,C,D,F).

To better understand the mouse brain inflammatory process following particle exposure, an in vivo analysis was performed by means of fluorescence molecular tomography (FMT) following injection of the MMPSenseTM 750 FAST probe, which becomes active and produces a fluorescent signal after cleavage by disease-related matrix metalloproteinases (MMPs), including MMP2, 3, 7, 9, 12, and 13. Twenty-four hours after a single intratracheal instillation, we found an increase in BB (+26.27 pmol)- and DEP (+10.09 pmol)-treated mice, when compared to sham (Figure 2G,H). Twenty-four hours after the last repeated intratracheal instillations, we observed no changes in DEP-treated mice, while BB-treated mice displayed an increase (+13.87 pmol), compared to sham (Figure 2I,J).

### 2.4. Evaluation of Amyloidogenic Precursor Protein Processing

To study AD-related neurodegeneration, amyloidogenic precursor protein (APP) and β-secretase (BACE1) protein levels were evaluated in sham and treated mice. Moreover, we analyzed APP phosphorylation on Thr668 (p-APP ^Thr668^), which was shown to enhance BACE1 proteolytic activity [36,37].

**Figure 3:**
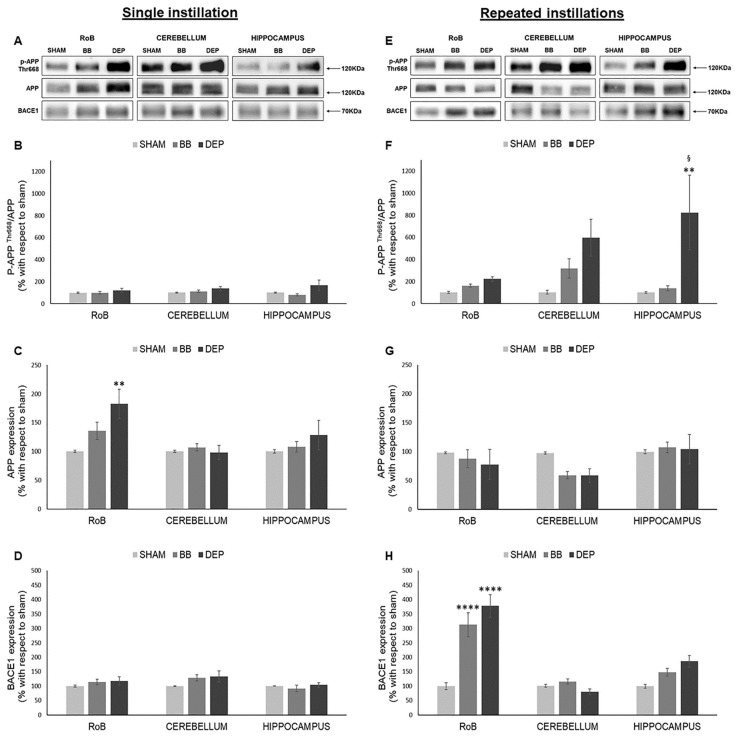
Amyloidogenic precursor protein (APP) processing analysis after single and repeated instillations of BB and DEP. Representative immunoblotting images of amyloid precursor protein (APP), phosphorylated APP on threonine 668 (p-APP ^Thr668^), and beta-secretase 1 (BACE1) analysis in mice after single (**A**) and repeated (**E**) instillations with 50 µg of BB or DEP/100 µL 0.9% NaCl. Histograms display p-APP ^Thr668^/APP, APP, and BACE1 protein levels in mice after single (**B**–**D**) and repeated (**F**–**H**) instillations with BB and DEP, with respect to sham. Proteins are normalized to corresponding total proteins revealed by Ponceau in each lane (Figure S3, Supplementary Materials), and the data are expressed as means ± SEM (*n* = 6). Statistical differences were tested accordingly by one-way ANOVA followed by Tukey post hoc comparison. ** *p* < 0.01 vs. sham mice; **** *p* < 0.0001 vs. sham mice; § *p* < 0.05 vs. BB-treated mice.

As shown in Figure 3, while after a single instillation we detected no variations in p-APP ^Thr668^/APP ratio, DEP repeated treatment induced its increase in all districts compared to sham: RoB (+121.46% ± 21.32%), cerebellum (+493.38% ± 166.87%), and hippocampus (+722.87% ± 337.57%). p-APP ^Thr668^/APP increase resulted statistically significant only in the hippocampus due to considerable variation between samples (Figure 3A,B,E,F). At the same time, RoB APP protein levels displayed a significant increase (+82.76% ± 25.53%) after DEP single instillation, while repeated instillations caused a decreasing trend in the RoB (−20.17% ± 12.62% with DEP) and cerebellum (−38.25% ± 12.17% with BB and −38.68% ± 11.80% with DEP) (Figure 3A,C,E,G).

Moreover, BACE1 protein levels showed no variations after a single instillation; after repeated exposure, our results suggest a strong significant increase in the RoB (+212.68% ± 41.26% with BB and +277.91% ± 39.07% with DEP) and an increasing trend in the hippocampus (+48.78% ± 13.23% with BB and +87.00% ± 19.90% with DEP), when compared to sham (Figure 3A,D,E,H).

## 3. Discussion

In this project, we analyzed and compared the effects of acute (single intratracheal instillation) and sub-acute (three intratracheal instillations in a week) BB and DEP peripheral administration on mouse brain regarding oxidative stress and inflammation induction, as well as their possible implications in brain pathologies. 

Alterations in some oxidative stress- and neuroinflammation-related markers were already found in rodents [38,39,40] and in vitro models [41,42,43] following diesel exhaust exposure. Moreover, most animal studies indicate that exposure to biomass particles can have a significant impact on the respiratory immune system [12] and induce oxidative stress, cytotoxicity, and genotoxicity [44]. However, little is known to consistently distinguish the toxicological effects of different types of UFPs. While a comparison between the toxicological effects of BB and DEP on the respiratory and cardiovascular systems was already published by our group [33], the novelty of this study is the comparison between their effects on different brain regions. In addition to oxidative stress and inflammation, neurodegeneration markers were analyzed to evaluate the UFP impact on neurodegenerative disease (e.g., AD). To this purpose, we analyzed different parts of the brain: the hippocampus, commonly involved in AD onset and progression, the cerebellum, generally not involved in AD neurodegeneration, and the remaining part of the brain (rest of brain, RoB).

To date, the literature proposes two principal routes to explain how UFPs may reach the brain. In one route, particles are considered to reach brain tissue following inhalation, deposition on the nasal olfactory epithelium, and translocation along the olfactory nerve. The other route involves alveolar deposition and subsequent crossing of the blood–air barrier and the BBB [3]. This second way includes the inflammatory mediators produced in the lungs because of pollutant-induced epithelial and endothelial injury that can be released into the circulation, reaching the brain [3,20]. UFP neuronal transport was also described, involving sensory nerves existing in the nasopharyngeal and tracheobronchial regions of the respiratory tract and, thus, circumventing the very tight BBB. In general, translocation rates of UFPs from the lung to the blood compartment or the CNS are much lower compared to nasal translocation. Important translocation modifiers are the physicochemical characteristics of particles, especially their size and surface chemistry [28]. We decided to perform intratracheal instillation, as a validated alternative method for aerosol inhalation toxicity testing. This technique is indeed able to generate reliable dose–response data; it is relatively easy to carry out, requiring a lower amount of particles and allowing shorter exposure times compared to conventional inhalation studies [45]. Thus, we suppose that particles or their components, together with inflammatory mediators produced in lungs, could reach the brain, inducing microglia phagocytic activity and leading to reactive oxygen species (ROS) production and possibly neuronal damage [46]. In this context, brain histopathological analyses evidenced that acute or sub-acute administration of BB and DEP did not affect brain morphology, while they induced the early activation of stress-related markers.

### 3.1. DEP Treatment Induces Higher Oxidative Stress Response Than BB in Mouse Brain

The brain is believed to be particularly vulnerable to oxidative stress, as it consumes relatively large amounts of oxygen during its metabolism, and it has lower antioxidant defenses compared to other organs [47]. Our analyses demonstrated that DEP particles were highly effective in inducing brain oxidative stress, while BB particles mostly produced protein expression increasing trends, without reaching statistical significance, as previously demonstrated for other tissues [10,33]. 

**Figure 4:**
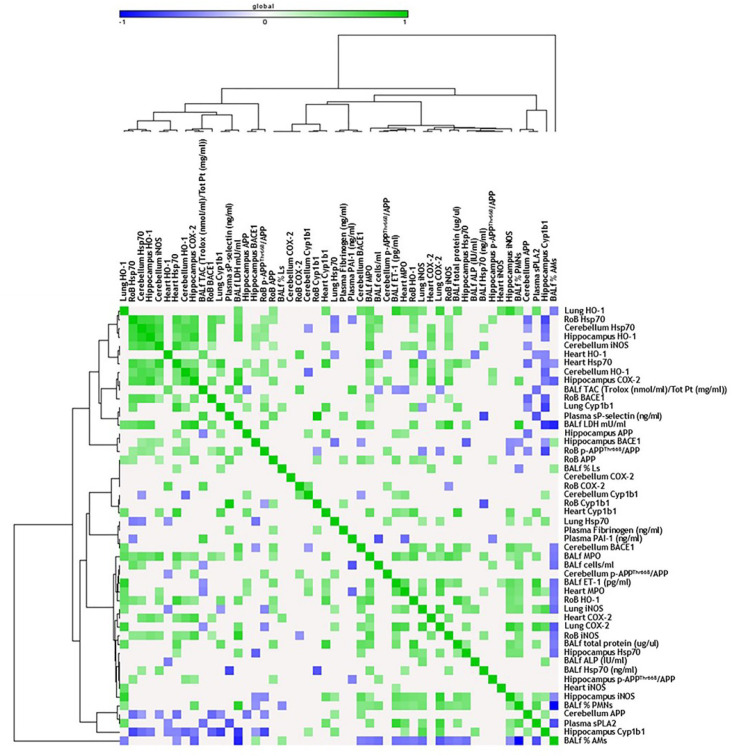
Correlation analysis in acute treatment. Heat map showing the Pearson correlation between markers expressed in the rest of brain (RoB), cerebellum, hippocampus, and respiratory and cardiovascular system markers assessed in the same animals and presented in our previous work [33], after a single instillation with 50 µg of BB or DEP/100 µL 0.9% NaCl. All the correlation data are reported in Table S1 (Supplementary Materials).

Consistent with the literature, acute DEP exposure induced HO-1 expression, especially in the RoB. HO-1 is a crucial enzyme for the antioxidant response and neuroprotection [48], and its increase in the RoB positively correlates with HO-1 and Cyp1b1 levels in the lung of the same mice [33]. Moreover, both the RoB and the hippocampus HO-1 levels positively correlate with the increase in heart parenchyma Hsp70 expression and bronchoalveolar lavage fluid (BALf) myeloperoxidase (MPO) levels [33], whose activity may be implicated in ROS generation [49] (Figure 4). These findings support the hypothesis of a widespread oxidative status induced after DEP particle deposition in the lungs and translocation of toxic components to the brain through systemic circulation. It is worth noting that, after sub-acute exposure, HO-1 levels seem to be restored, suggesting the establishment of some compensatory mechanisms in order to limit the toxic reaction generated after a single treatment. These data are in line with Kim et al. [50], who showed that HO-1 protein levels are not significantly altered in different brain regions after long-term PM inhalation.

**Figure 5:**
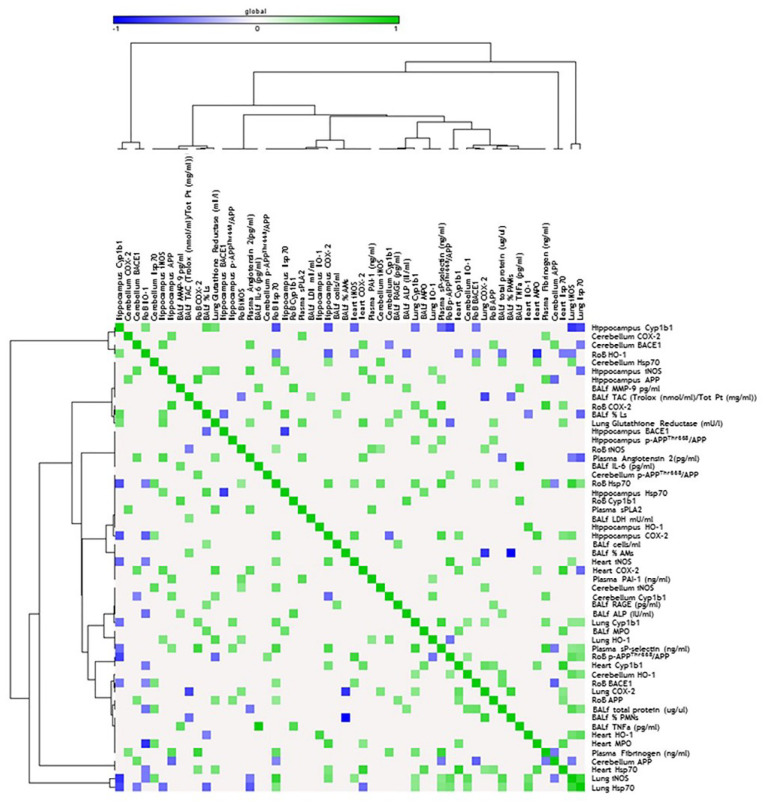
Correlation analysis in sub-acute treatment. Heat map showing the Pearson correlation between markers expressed in the RoB, cerebellum, hippocampus, and respiratory and cardiovascular system markers assessed in the same animals and presented in our previous work [33], after repeated instillation with 50 µg of BB or DEP/100 µL 0.9% NaCl. All the correlation data are reported in Table S1 (Supplementary Materials).

The oxidative stress condition in our model was also confirmed by the increase of Hsp70, a heat-shock protein that plays a key role in preserving normal cell function after different insults and whose modulation was described in the literature in DEP-induced oxidative stress conditions [51]. After acute DEP exposure, we observed a significant increase in Hsp70 levels in the RoB, which positively correlates with several markers, including HO-1 and iNOS expressed in the same brain areas, and Hsp70 and MPO increased in the heart and BALf [33]. The increased expression of Hsp70 was maintained after sub-acute DEP exposure in all brain parts and was positively correlated with different oxidative stress and inflammation markers expressed in the heart of the same mice [33] (Figure 5). DEP is rich in transition metals such as Zn and V, which could be responsible for the DEP-induced Hsp70 increase, as already demonstrated by Graff and collaborators [52]. Furthermore, compared to BB, DEP contains a higher amount of PAHs, which were described as inducers of Hsp70 expression in various experimental systems [53]. 

PAHs adsorbed on the surface of DEP particles are known to dissolve and translocate into blood circulation after particle deposition in the lungs [54], mostly linked to and transported by albumin [55]. However, it was demonstrated that the number of particles and, hence, the eventual concentration of particle-bound PAHs and other organic constituents that may reach the brain within 6 h after DEP exposure would be rather low [2]. In agreement with this, we observed that, after acute DEP exposure, brain Cyp1b1 resulted unchanged. On the contrary, sub-acute DEP exposure significantly increased Cyp1b1 in the RoB and cerebellum, compared to sham and BB-treated mice, while the hippocampus showed a non-significant rise. Thus, we hypothesized that DEP particles or PAHs may reach the brain after sub-acute administration. In the brain, they may be converted into reactive oxygenated PAH intermediates capable of interacting with cellular macromolecules, sustaining oxidative stress damage. It is worth mentioning that a positive correlation between the increased expression of Cyp1b1 in cerebellum and heart was observed.

### 3.2. Both BB and DEP Treatment Induces Inflammation in Mouse Brain

The literature describes the presence of inflammatory markers, including iNOS and COX-2, in the brain of humans and animals exposed to high levels of air pollution [56,57,58,59]. In agreement with the results obtained in respiratory and cardiovascular systems [10,33], our analyses demonstrated that BB and DEP differently induce inflammation-related mechanisms in mouse brain. While BB exposure was not very effective in iNOS stimulation, we observed that acute DEP administration induced the expression of iNOS in the hippocampus, with induction detected after sub-acute exposure in all the considered brain regions. It is worth mentioning that iNOS expression in sham 3 h after a single instillation with saline solution was higher when compared with the repeated exposure analysis, probably because of the mechanical procedure, which induces a low level of inflammation even without particles.

The iNOS increase after UFP instillation was positively correlated with oxidative stress and inflammation markers expressed in other tissues, especially after a single exposure. Interestingly, sub-acute iNOS expression was positively correlated with Hsp70 expression in the respective areas, confirming the existence of a relationship already described in the literature [60,61]. Therefore, we could hypothesize that DEP peripheral administration favors nitric oxide (NO) production in mouse brain. NO can be rapidly oxidized under aerobic conditions to reactive nitrogen oxide species (RNOS), unstable molecules that, at high concentrations, can induce cell toxicity by nitrosating DNA and protein and by inducing lipid peroxidation [62]. 

The presence of an inflammatory status after acute treatment was also demonstrated by COX-2 expression in the RoB and hippocampus with both BB and DEP. Notably, we found positive correlations between COX-2 expression in the hippocampus and other oxidative stress and inflammatory marker expression in the heart and BALf [33]. While iNOS expression was greater after sub-acute treatment compared to acute exposure, COX-2 induction was significantly maintained after repeated instillation only in the RoB of DEP-treated mice, suggesting the existence of a recovery mechanism [63]. These data are in contrast with preliminary analyses performed by Bhatt and collaborators [64], which showed COX-2 expression in the brain even after nine months of PM inhalation. 

To further investigate the effect of BB and DEP intratracheal instillation, we tested in vivo the inflammatory status, taking advantage of FMT and analyzing the activation of brain MMPs, a family of proteolytic enzymes involved in physiological and pathological conditions [65]. Our examinations demonstrated that exposure to traffic-generated air pollutants promotes the increase of MMP activity, which could result in degradation of tight junction proteins in the cerebral vasculature, alteration of BBB permeability, and expression of neuroinflammatory markers [66]. Our FMT results displayed activation of MMPs 24 h after a single instillation with both BB and DEP, while, 24 h after repeated exposure, only BB-treated mice showed MMP activation. Notably, while the stimulation on brain MMPs after diesel exposure was recently reported in the literature [67], there are no available data regarding their activation by biomass particles. However, it is possible to speculate that, since BB is enriched in Mn, a known MMP inductor [68], specific metal and trace element concentrations may have a key role in the stimulation of different pathways along the time.

Thus, our results suggest that inflammation is induced in mouse brain after acute peripheral administration of both UFPs as a protective mechanism. On the contrary, the existence of chronic inflammation that may occur in lifetime exposure is believed to be an important component contributing to AD progression [64].

### 3.3. UFP Treatment Induces Changes of AD-Related Proteins in Mouse Brain

Literature data suggest that air pollution-derived chemicals and organic compounds may be implicated in AD pathogenesis [42,69,70,71], with particular attention paid to the involvement of oxidative stress induction and inflammatory changes [34,35,57]. Interestingly, brain APP amyloidogenic processing was observed after long-term PM exposure in both humans [35] and mice [64]. APP is a transmembrane glycoprotein recognized and cleaved by a family of secretases, which work in pairs on specific amino-acid sequences. APP amyloidogenic processing is defined as the combined action of β-secretase (BACE1) and γ-secretase [72,73]. 

Our analyses evidenced no differences in APP and BACE1 protein levels after acute exposure to both BB and DEP. The only exception was the APP increasing trend in the RoB of treated mice, which was probably a compensatory mechanism activated after particle exposure, since the literature suggests that APP has a trophic function [74]. It is also interesting to note that the APP increasing trend in RoB is positively correlated to oxidative stress marker expression in the same brain district (HO-1, Cyp1b1, and Hsp70), as well as in the lung (HO-1) and BALf (MPO) of the same animals [33]. 

On the other hand, sub-acute BB and DEP exposure seems to affect brain APP processing, as we found a decreasing trend of APP expression in the RoB and cerebellum, especially after DEP exposure. This processing might depend on BACE1, whose protein levels were highly increased in the RoB of BB- and DEP-treated mice. Surprisingly, hippocampus APP levels resulted unchanged, despite BACE1 expression showing an increasing trend. 

In addition, the regulation of APP function and metabolism depends on post-translational modifications such as phosphorylation on Thr668 (numbering for APP695 isoform), predominantly observed in AD brain [36]. p-APP ^Thr668^, mediated by kinases such as glycogen synthase kinase-3 (GSK-3), c-Jun N-terminal Kinase (JNK), extracellular signal-regulated kinase (ERK), cyclin dependent kinase 5 (cdk5), cyclin dependent kinase 4 (cdk4), and cyclin dependent kinase 1 (cdc2), i.e., proteins activated in compromised neurons [75], was shown to enhance proteolysis by β-secretase [36,37]. Notably, to the best of our knowledge, p-APP ^Thr668^ was never described in relation to PM exposure. Our results suggest that DEP sub-acute exposure mainly induces an increase of the p-APP ^Thr668^/APP ratio in different parts of the mouse brain, which resulted statistically significant only in the hippocampus, probably because of considerable variation between the samples. However, the meaning of these post-translational modifications after UFP exposure remains to be investigated. 

In conclusion, we demonstrated that APP and BACE1 protein levels undergo some alterations after BB and DEP sub-acute exposure, and we hypothesize that these changes may become more prominent during chronic exposure, as demonstrated by mouse long-term PM inhalation performed by Bhatt and collaborators [64]. UFP continuous exposure, together with other factors like diabetes, hyperlipidemia, hypertension, heart disease, obesity (defined as noncommunicable diseases), and smoking [76], could represent a risk factor for AD onset and progression.

## 4. Materials and Methods 

### 4.1. Materials

All commercial chemicals were of the highest available grade. All powdered reactants and solutions for electrophoresis were from Sigma Chemical Co. (Milano, Italy). All the stock solutions for cell culture were from Euroclone (Celbio Milano, Italy). Precision Plus Protein Standards (All Blue) were from Bio-Rad (Milano, Italy) (Cat# 1610373). Complete protease inhibitor cocktail was from Roche Diagnostics S.p.A (Milano, Italy) (Cat# 11836145001). All the reagents used for histopathological analyses were from Bio-Optica (Milano, Italy).

### 4.2. Animals

Male BALB/cOlaHsd mice (7–8 weeks) were purchased from Envigo (San Pietro al Natisone, Udine, Italy) and housed in plastic cages under controlled environmental conditions (temperature 19–21 °C, humidity 40–70%, lights on from 7:00 a.m. to 7:00 p.m.), where food and water were administered ad libitum. Animal use and care procedures were approved by the Institutional Animal Care and Use Committee of the University of Milano-Bicocca (institutional application 002/2014) and complied with the guidelines set by the Italian Ministry of Health (approval number 381/2015-PR) (DL 26/2014). The study was not pre-registered. Invasive procedures were performed under anesthesia, and all efforts were made to minimize animal suffering.

### 4.3. UFPs Characterization

The UFPs employed in this work were provided by Camatini’s group, which used the same batch of UFPs to analyze their effects on human bronchial epithelial (HBEC3) cells [10]. Briefly, BB particles were sampled from a modern automatic 25-kW boiler, propelled by a prime quality spruce pellet, while DEP was collected from a Euro 4 light-duty vehicle without DPF, fueled by commercial diesel and run over a chassis dyno. Transmission electron microscopy (TEM) and scanning electron microscopy (SEM) images of both BB and DEP samples showed aggregates of round carbonaceous particles lower than 50 nm in diameter. In addition, DEP showed a higher PAH and transition metal (Fe, Zn, Cr, Pb, V, and Ni) concentration compared to BB, which conversely resulted in it being enriched in elements typical of wood combustion, such as Mn, K, and S (Table 1). The details of the complete UFP composition were already published [10].

### 4.4. Intratracheal Instillation 

Animal testing was carried out in the morning in the housing facility. Animals were randomly divided into three experimental groups for exposition to the different UFPs: sham (isotonic solution), BB-treated mice, and DEP-treated mice. Three mice for each experimental group were intratracheally instilled, and the experiments were replicated twice, for a total of six sham, six BB-treated, and six DEP-treated mice. The sample size is in line with previous published data on the adverse effects of air pollutants [33,58,77] with the aim of minimizing the number of animals employed. Mice, numbered 1–9 in each experiment, were exposed to a mixture of 2.5% isoflurane (Flurane, Merial, Toulouse) anesthetic gas and kept under anesthesia for the whole instillation procedure. Just before the intratracheal instillation, BB and DEP aliquots were diluted in sterile saline, sonicated, and then immediately instilled in mice. Intratracheal instillations with 50 µg of BB or DEP in 100 µL of isotonic saline solution or with 100 µL of isotonic saline solution [11] were achieved by means of a MicroSprayer^®^ Aerosolizer system (MicroSprayer^®^ Aerosolizer- Model IA-1C and FMJ-250 High Pressure Syringe, Penn Century, USA; validated by Bivas-Benita and collaborators [78]), as previously described [33,79,80,81]. The dose of 50 µg of BB or DEP in 100 µL of isotonic saline solution was selected as the best dose to test the effects of acute and sub-acute UFPs treatment, as suggested by Kaewamatawong et al. [82]. This dose was the same as the one we used in a parallel work [33], similarly to other in vivo investigations reported in the literature [21,83].

Immediately following instillation, treated and sham mice were allowed to recover under visual control and kept under controlled environmental conditions.

**Figure 6:**
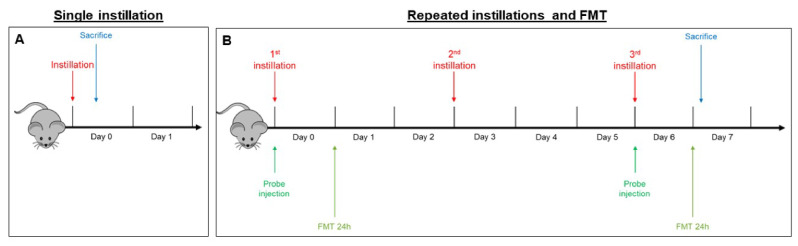
Schematic representation of BALB/c mice acute (**A**) and sub-acute (**B**) treatment and fluorescence molecular tomography (FMT).

Two different exposures were performed:

- In the acute exposure setting, mice were euthanized 3 h following a single instillation [33,58] in order to analyze early mechanisms of neurotoxicity, i.e., oxidative stress and inflammation (Figure 6A).
- In the sub-acute exposure setting, the intratracheal instillation was performed on days 0, 3, and 6, for a total of three instillations. Mice were euthanized 24 h following the last instillation [33,84,85], to understand if the effect of the single individual instillation was amplified when repeated or if a normal condition was restored (Figure 6B).

### 4.5. Fluorescent Molecular Tomography

Fluorescence molecular tomography (FMT^®^) is an imaging technique for improved localization and quantification of fluorescent probes in deep tissue. Two male BALB/c mice were injected intravenously with MMPSenseTM 750 FAST probe (PerkinElmer, Waltham, MA, USA) (Cat# NEV10168) immediately before the first instillation (day 0). This probe is an MMP (metalloproteinase)-activatable agent that produces a fluorescent signal after cleavage by disease-related MMPs, including MMP2, 3, 7, 9, 12, and 13. FMT examination 3 h after a single instillation was not possible because the recommended optimal imaging time point was 6–24 h post injection of the probe, to allow its distribution and the decrease of background signal. Twenty-four hours after probe injection, anesthetized mice were depilated and placed into the imaging cassette inside the chamber of the FMT 1500TM In Vivo Imaging System (PerkinElmer, Milan, Italy). This procedure was repeated at day 6, 24 h after the last repeated instillation (Figure 6B). 

The total amount of fluorophore (in picomoles) in a selected three-dimensional region of interest (ROI) was calculated by the TrueQuant software, version 3.1 [86] (PerkinElmer, Milan, Italy). In order to abolish any operator bias, ROIs were drawn by an operator blinded to the experimental origin of the specimens.

### 4.6. Brain Dissection 

Male BALB/c mice were exposed to a mixture of 2.5% isoflurane (Flurane) anesthetic gas. When a deep state of anesthesia was achieved, cervical dislocation was performed. The dissection of neuronal tissues was performed on fresh brain based on visual information and on the natural anatomical boundaries of certain regions present in the brain [87]. After removing the brainstem, the left hemispheres were collected for histological analysis, while the right hemispheres were further dissected for Western blot. The cerebellum was detached, and the hippocampus was extracted as described by Hagihara and collaborators [88]. Then, the cerebellum, hippocampus, and the remaining part of the brain (rest of brain, RoB) were placed in isotonic saline solution. 

### 4.7. Tissues Homogenization

The size of the RoB and cerebellum allows their mechanical homogenization using a Potter tissue grinder. For this purpose, each piece was suspended in 1:15 (*w*/*v*) sterile isotonic saline solution plus protease and phosphatase inhibitors. The samples were then sonicated and centrifuged at 18,400× *g* for 10 min at 4 °C, and the supernatants were transferred to new tubes. Subsequently, the appropriate volume of radioimmunoprecipitation assay buffer (RIPA) buffer 5× (125 mM Tris-HCl pH 7.4, 750 mM NaCl, 5% NP40, 2.5% C_24_H_40_O_4_, 0.5% SDS, plus protease inhibitor cocktail and phosphatase inhibitors) was added in order to ensure an efficient cell lysis for SDS-PAGE. On the other hand, the size of the hippocampus does not allow its mechanical homogenization using a Potter tissue grinder. Therefore, 100 µL of RIPA buffer 1× was added to each hippocampus. The samples were left on ice for 5 min, sonicated, and stored at −20 °C for subsequent biochemical analyses. All the above procedures were performed on ice.

### 4.8. Histopathological Analyses 

For histopathological examinations, the left brain hemisphere of sham and treated mice was quickly harvested, washed with 0.1 M phosphate-buffered saline (PBS, pH 7.4), fixed in 10% neutral buffered formalin for 48 h, and paraffin-embedded (Embedding Center Leica EG1160, Leica Biosystems, Milan, Italy) after ethanol-based dehydration. Sections (4 μm thick) were cut with a rotary microtome (Leica RM2255, Leica Biosystems, Milan, Italy), stained with hematoxylin and eosin (H&E) (BIO-OPTICA, Cat# 05-M06002, 05-M10002) and evaluated under a light microscope (Nikon Eclipse 50i, Melville, NY, USA). Representative images were captured with a digital camera (Nikon Digital Sight DS-2Mv, Melville, NY, USA). Histopathological analyses were performed by an operator blinded to the experimental origin of the specimens.

### 4.9. SDS-PAGE Electrophoresis and Immunoblotting

The RoB, cerebellum, and hippocampus homogenates were subjected to protein quantification with a micro-bicinchoninic acid (BCA) assay (Sigma-Aldrich Cat# B9643, Cat# C2284); then, 30 µg of total proteins for each sample were subjected to SDS-PAGE (10%) followed by Western blot. Immunoblottings were performed using anti-rabbit polyclonal APP (CT695) (1:500) (Thermofisher Scientific™ Cat# 51-2700, RRID:AB_2533902), anti-rabbit monoclonal phospho Thr668 APP (p-APP ^Thr668^) (1:1000) (Cell Signaling Technology Cat# 6986, RRID:AB_10831197), anti-rabbit monoclonal BACE1 [EPR3956] (1:2000) (Abcam Cat# ab108394, RRID:AB_10861218), anti-rabbit polyclonal cyclooxygenase-2 (COX-2) (1:1000) (Cell Signaling Technology Cat# 4842, RRID:AB_2084968), anti-rabbit polyclonal cytochrome 1b1 (Cyp1b1) (1:800) (Santa Cruz Biotechnology Cat# sc-32882, RRID:AB_2089112), anti-rabbit polyclonal heme oxygenase-1 (HO-1) (1:200) (Santa Cruz Biotechnology Cat# sc-10789, RRID:AB_648281), anti-goat polyclonal Hsp70 (1:200) (Santa Cruz Biotechnology Cat# sc-1060, RRID:AB_631685), and anti-rabbit polyclonal inducible nitric oxide synthase (iNOS) (1:400) (Santa Cruz Biotechnology Cat# sc-8310, RRID:AB_2152867) antibodies. The secondary antibodies were appropriate horseradish peroxidase (HRP)-conjugated goat anti-rabbit (1:5000) (Thermo Fisher Scientific Cat# 31460, RRID:AB_228341) or donkey anti-goat (1:2000) (Santa Cruz Biotechnology Cat# sc-2020, RRID:AB_631728). Immunoreactive proteins were revealed by enhanced chemiluminescence (ECL) and semi-quantitatively estimated by ImageQuant™ LAS 4000 (GE Healthcare Life Sciences, Milan, Italy). No blinding was performed.

Recent studies reported that total protein staining represents the actual loading amount more accurately than a housekeeping protein due to minor procedural and biological variations [89,90]. In addition, variability of housekeeping genes was observed in neuronal diseases or brain injury conditions [89]. Thus, samples were normalized with respect to the total amount of proteins detected by Ponceau staining, allowing a straightforward correction for lane-to-lane variation [89,90,91].

### 4.10. Statistical Analysis 

Data were assembled with Galaxy [92] and analyzed with the IBM SPSS Statistical Package v25 (IBM, Armonk, NY, USA) by an operator blinded to the experimental origin of the specimens. No exclusion criteria were pre-determined. Linear regression analyses were performed by Pearson correlation. For each parameter measured in sham, BB-treated, and DEP-treated mice, the means (± standard error of the mean, SEM) were calculated by ANOVA with Tukey multiple-comparison post hoc correction. A significance threshold of *p* < 0.05 was used. 

## 5. Conclusions

In summary, as previously demonstrated for other tissues [10,33], DEP exposure was more harmful than BB in brain tissue. Indeed, although both acute exposures induced inflammatory-related pathways, only DEP showed a strong oxidative stress activation. The sub-acute exposure sustains these stress-related processes and additionally induces the modulation of APP and BACE1 protein levels for both UFPs. Moreover, among the three different brain regions considered in this work, RoB resulted as the most affected by a single instillation, especially by DEP exposure. On the other hand, repeated instillations resulted in variations of oxidative stress and inflammation affecting all observed brain districts.

Considerable variation in the responses to both diesel and biomass particles was already described in the literature [31,93]. Specifically, DEP also resulted highly effective in in vitro experiments performed by Longhin and collaborators using the same particles, while BB treatment was mostly ineffective [10], indicating that the different UFP chemical composition is the key to the differential stress response. 

In conclusion, our results suggest that both acute and sub-acute UFP peripheral administration can induce CNS response. Whether this activation is due to the direct transport of UFPs or inflammatory mediators to the brain remains to be investigated, although our hypothesis is that, in our experimental conditions, UFPs do not directly translocate to the brain, and the effects observed are most likely due to the translocation of inflammatory mediators and/or toxic components.

## Figures and Tables

**Table 1:** Chemical compositions of ultrafine particles (UFPs) from different anthropogenic sources, as reported in Longhin et al., 2016. [10]. Each value is expressed as mean concentration (±SD). DEP—diesel exhaust particles; BB—biomass burning-derived particles; PAH—polycyclic aromatic hydrocarbon; ND—not determined.

Element	Unit	DEP	BB
Al	ng/µg	135 ± 4	ND
K	ng/µg	50 ± 0.02	195 ± 12.5
Ca	ng/µg	198 ± 8	70 ± 4
Fe	ng/µg	4 ± 0.001	ND
Zn	ng/µg	70 ± 2	4 ± 0.001
Cr	ng/µg	0.04 ± 0.001	ND
Mn	ng/µg	0.03 ± 0.001	0.42 ± 0.03
V	ng/µg	0.05 ± 0.007	ND
Ni	ng/µg	0.02 ± 0.001	ND
Pb	ng/µg	0.02 ± 0.001	ND
Total PAHs	ng/mg	600 ± 150	50 ± 10

## Supplementary Materials

Supplementary materials can be found at https://www.mdpi.com/1422-0067/21/10/3699/s1.
