# Supplementary material for: Systemic Exposure to Air Pollution Induces Oxidative Stress and Inflammation in Mouse Brain, Contributing to Neurodegeneration Onset
Source: Int J Mol Sci. 2020 May 24;21(10):3699. doi: 10.3390/ijms21103699 (PMC7279458; doi:10.3390/ijms21103699)
Supplement: Supplementary file 1 [file ijms-21-03699-s001.zip › ijms-777213 suppl/SUPPLEMENTARY MATERIAL.pdf]

SUPPLEMENTARY MATERIAL

Systemic Exposure to Air Pollution Induces Oxidative Stress and Inflammation in Mouse Brain, Contributing to Neurodegeneration Onset

Chiara Milani [1,3,*], Francesca Farina [1,3,4], Laura Botto [1,3,4], Luca Massimino [5], Elena Lonati [1,3,4], Elisabetta Donzelli [1,3], Elisa Ballarini [1,3], Luca Crippa [1,3], Paola Marmiroli [2,3], Alessandra Bulbarelli [1,3,4] and Paola Palestini [1,3,4]

[1] School of Medicine and Surgery, University of Milano-Bicocca, 20900 Monza, Italy; francesca.farina@hotmail.it (F.F.); laura.botto@unimib.it (L.B.); elena.lonati1@unimib.it (E.L.); elisabetta.donzelli@unimib.it (E.D.); elisa.ballarini@unimib.it (E.B.); luca.crippa@unimib.it (L.C.); alessandra.bulbarelli@unimib.it (A.B.); paola.palestini@unimib.it (P.P.)

[2] Department of Biotechnology and Biosciences, University of Milano-Bicocca, 20126 Milan, Italy; paola.marmiroli@unimib.it (P.M.)

[3] NeuroMi, Milan Centre for Neuroscience, University of Milano-Bicocca, 20900 Monza, Italy

[4] POLARIS Research Centre, University of Milano-Bicocca, 20126 Milan, Italy

[5] Division of Neuroscience, San Raffaele scientific institute, 20121 Milan, Italy; admin@lucamassimino.com (L.M.)

* Correspondence: chiara.milani@unimib.it (C.M.); Tel.: +39-02-6448-8089; Fax: +39-02-64488068

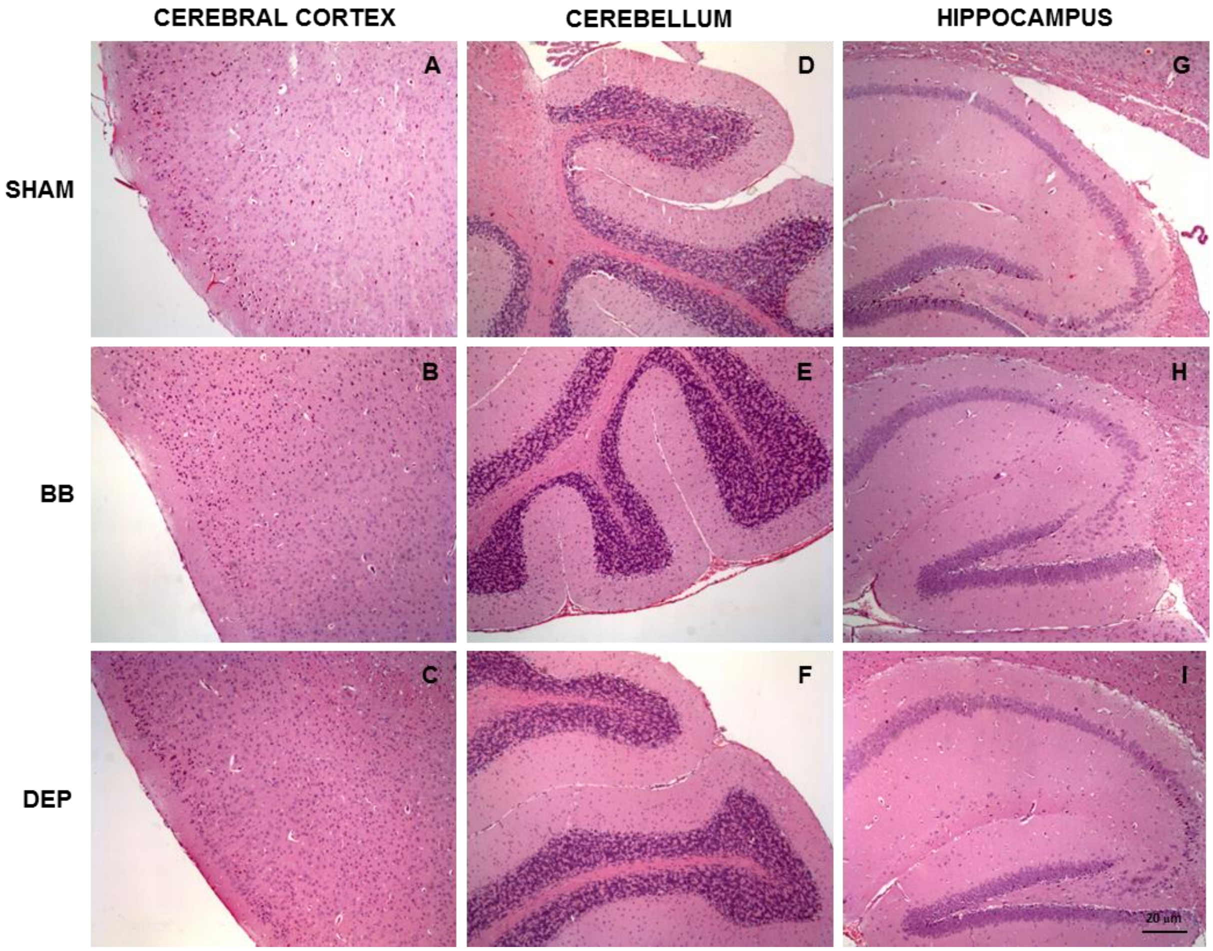

Figure S1: **Histopathological analyses of brain tissue after single instillation of BB and DEP**. Representative histological images of cerebral cortex **(A, B, C)**, cerebellum **(D, E, F)** and hippocampus **(G, H, I)** after a single intratracheal instillation with 50µg of BB or DEP/100µl 0.9% NaCl. Each figure represents the status evidenced examining 2 sham and treated mice. Scale bar=20 µm.

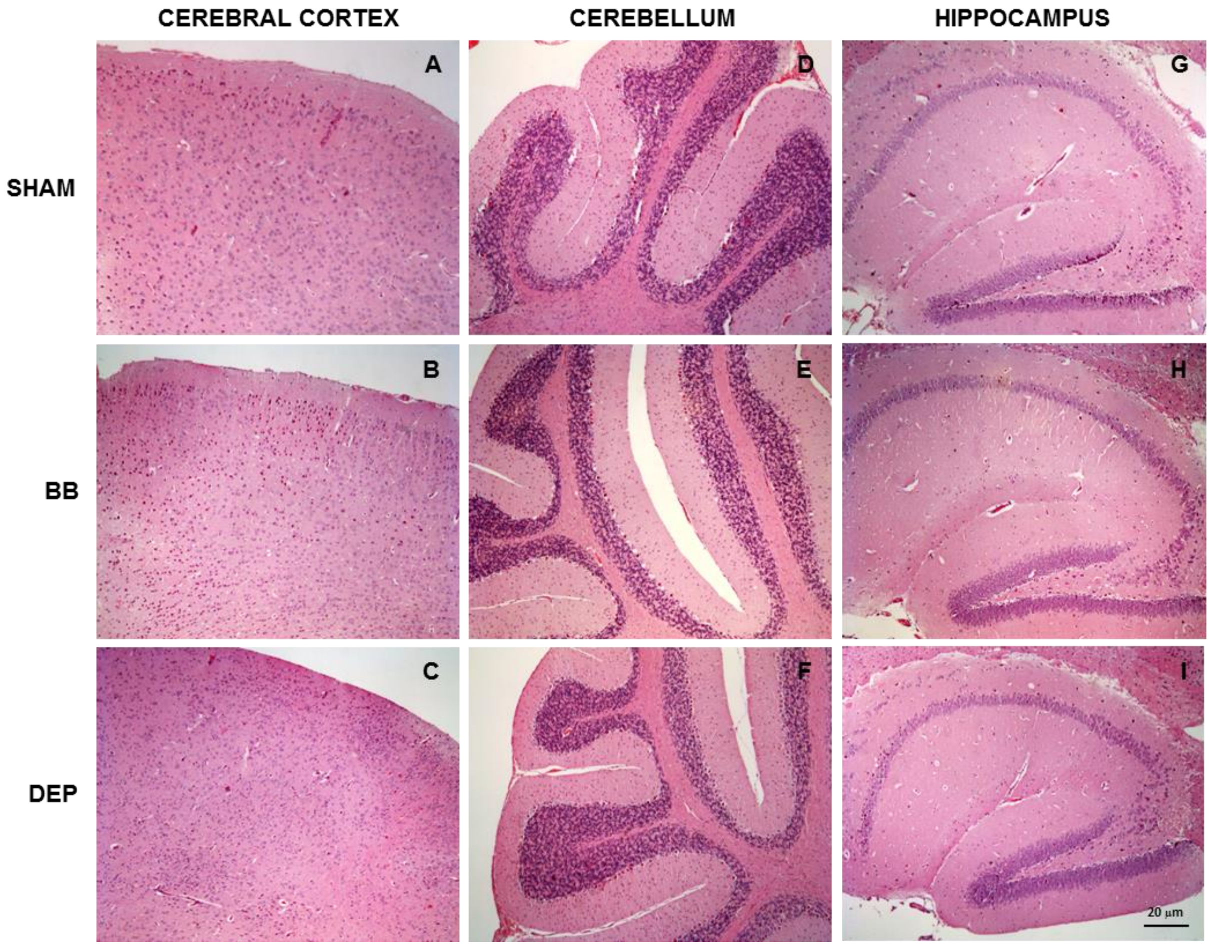

Figure S2: **Histopathological analyses of brain tissue after repeated instillations of BB and DEP**. Representative histological images of cerebral cortex **(A, B, C)**, cerebellum **(D, E, F)** and hippocampus **(G, H, I)** after repeated intratracheal instillations with 50µg of BB or DEP/100µl 0.9% NaCl. Each figure represents the status evidenced examining 2 sham and treated mice. Scale bar=20 µm.

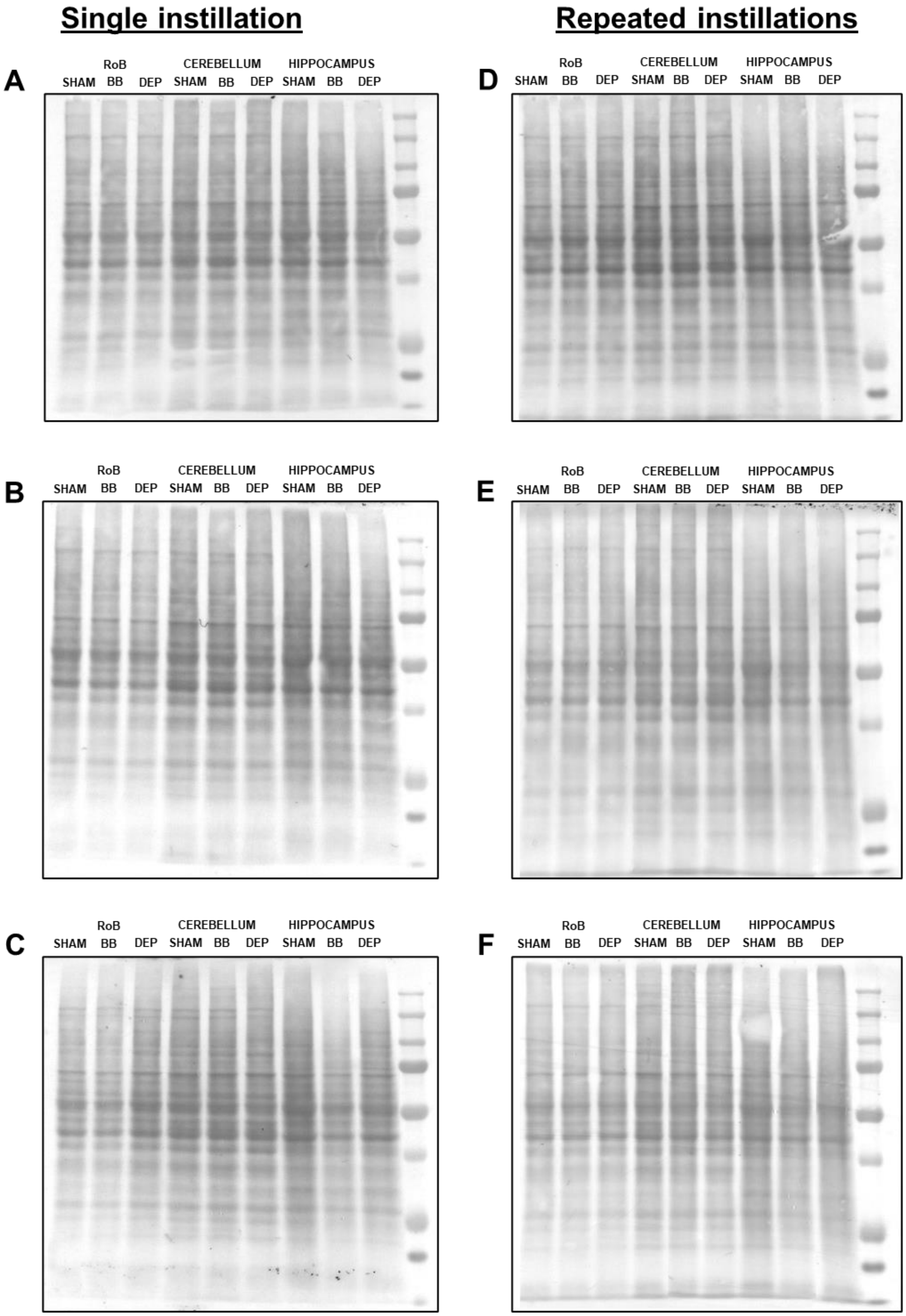

Figure S3: **Ponceau staining.** Representative Ponceau staining for western blot protein normalization obtained after single and repeated intratracheal instillations with 50µg of BB or DEP/100µl 0.9% NaCl. **(A, D)** Figure 1. **(B, E)** Figure 2. **(C, F)** Figure 3.
